# Diagnosis and treatment of adrenocorticotrophic hormone-independent macronodular adrenocortical hyperplasia: A report of 23 cases in a single center

**DOI:** 10.3892/etm.2014.2115

**Published:** 2014-12-08

**Authors:** JIANG LI, CHANG-HAI YANG

**Affiliations:** Department of Urology, Tianjin Medical University General Hospital, Tianjin 300052, P.R. China

**Keywords:** adrenocortical macronodular hyperplasia, Cushing’s syndrome, patients

## Abstract

The aim of the present study was to present the treatment observations of patients with adrenocorticotrophic hormone (ACTH)-independent macronodular adrenal hyperplasia (AIMAH) from a single medical center. A total of 23 patients with AIMAH were retrospectively reviewed according to the clinical features of the patient. Physical symptoms and biochemical evidence of Cushing’s syndrome (CS) were present in all the cases. High and low dose dexamethasone failed to suppress cortisone secretion on the suppression tests and ACTH levels were low in all the cases. Bilateral enlarged adrenal glands were observed with computed tomography scans in the 23 cases, which were determined to be bilateral adrenal macronodular hyperplasia by pathological analysis. Normal blood pressure was observed in eight patients in the 2–8 years following the bilateral adrenalectomy. A total of 15 cases underwent single side adrenalectomy and their blood pressures markedly decreased three years following surgery; medical treatment was administered to 12 of the 15 patients, while three patients underwent a contralateral adrenalectomy. Nelson’s syndrome was not diagnosed for any of the cases. AIMAH has unique endocrinological, radiological and pathological features, presenting as an independent etiological factor of CS. Diagnosis of AIMAH is usually derived from pathological examination and long term remission may be achieved by unilateral adrenalectomy. Contralateral adrenalectomy should be performed in cases of recurrence, when followed with periodical examination of the symptoms and the serum concentration of cortisol.

## Introduction

Adrenocorticotrophic hormone (ACTH)-independent macronodular adrenal hyperplasia (AIMAH) is a rare disorder characterized by bilateral macronodular hyperplasia of the adrenal glands and increased cortisol production with subclinical or overt Cushing’s syndrome (CS) (1,2). AIMAH represents <1% of cases of endogenous CS; however, since 10% of adrenal lesions are bilateral, AIMAH with subclinical cortisol secretion is becoming increasingly recognized (3). Patients with AIMAH are diagnosed following incidental radiological observations or the investigation of an adrenal over-secretion syndrome (4,5). AIMAH usually presents without symptoms, although in a number of cases patients are diagnosed following clinical CS.

The most common cause (95% of patients) of AIMAH is adrenocortical adenoma or carcinoma. The majority of the remaining patients have primary pigmented nodular adrenal disease, a syndrome that is characterized by multiple small bilateral pigmented adrenocortical nodules, and is often associated with the Carney complex (6). The diagnosis and management of patients with ACTH-independent CS and bilateral adrenal masses are problematic (7,8), particularly for bilateral adrenal adenomas. In the current study, a total of 23 AIMAH cases are presented; the patients were admitted to the Tianjin Medical University General Hospital (Tianjin, China) between July 1994 and 2010, and diagnosed by pathology.

## Materials and methods

### Patients

The present study was approved and registered by the Ethics Committee of the Tianjin Medical University General Hospital (Tianjin, China) in January 1993. The Ethics Committee approved all associated screening, treatment, data collection and follow-up of the patients; written informed consent was obtained from all particpants. All experiments were undertaken in accordance with the Declaration of Helsinki. A total of 23 patients (males, 14; females, 9; mean age, 49 years) were admitted to the Tianjin Medical University General Hospital between July 1994 and 2010. All the patients presented with several symptoms characteristic of CS, and hypertension was observed during examination. The duration of the disease ranged between one and five years. Diabetes occurred in 10 cases (10/23), central obesity occurred in eight cases, a sanguine appearance was observed in six cases and 14 patients exhibited purple stripes on their body.

The levels of plasma cortisol, ACTH and urinary free cortisol (UFC) were analyzed in all the patients using DPC Immulite 2000 (Siemens Healthcare, Los Angeles, CA, USA), and patients were subjected to high and low dose dexamethasone suppression tests (HDDST and LDDST). Plasma cortisol levels were monitored in these patients, Magnetic resonance imaging (MRI) examinations (Discovery MR750w 3.0T; GE Healthcare, Pittsburgh, PA, USA) and computed tomography (CT) scans (Lightspeed VCT XT; GE Healthcare) were also performed.

### Operative procedures

Bilateral adrenalectomy was performed in eight patients and unilateral adrenalectomy was performed in 15 patients. The surgical procedures were performed as previously described by Shinbo *et al* (9). Briefly, under general anesthesia, patients undergoing a right adrenalectomy were placed in a left lateral position. Next, 10/12-mm trocars were placed at the mid-clavicular line below the costal margin and at the median line 5 cm above the umbilicus, while 5-mm trocars were placed at approximately three finger-widths below the xyphoid process and at the anterior axillary line 5 cm below the costal margin. Laparosonic coagulating shears with a suction and irrigation device and with a cautery and L-hook tip were used when necessary. The two trocars at the median line remained on the abdominal wall while skin wounds at the other trocar sites were closed.

For left laparoscopic adrenalectomy, patients were repositioned in a right half lateral position, and two 5-mm trocars were placed at the left region of the costal margin and at the midclavicular line below the costal margin. The left adrenal gland was isolated similarly to right side and enclosed in an endoscopic pouch. Skin wounds were closed.

## Results

### Laboratory testing results

Results of the HDDST and LDDST were negative. Four patients received plasma cortisol rhythm determination, while the plasma cortisol levels in the additional 19 cases were only examined at 8:00 am. The results demonstrated that plasma cortisol levels were elevated in 20 patients. In addition, the levels of UFC were increased, while the levels of ACTH were decreased in the 23 patients (Table I).

### Imaging examination

**Figure 1:**
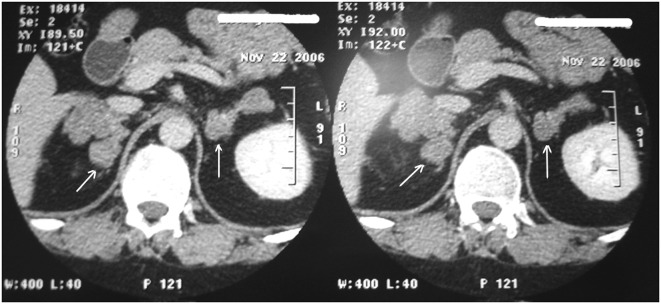
CT scan of one patient, whose bilateral adrenal gland demonstrated irregular nodular masses following enhancement. CT, computed tomography.

MRI examinations revealed that the pituitary gland was normal in 17 patients; however, MRI of the pituitary gland was not performed in the remaining six patients. Observations from the CT scans revealed bilateral adrenal nodules of soft tissue density, measuring ≤5 cm, and irregular nodular masses in the adrenal glands. In addition, the CT scans demonstrated that the adrenal lesions with macronodularity were significantly enlarged (Fig. 1), and the largest diameter of an adrenal nodular was 6 cm.

### Surgery and discharge

**Figure 2:**
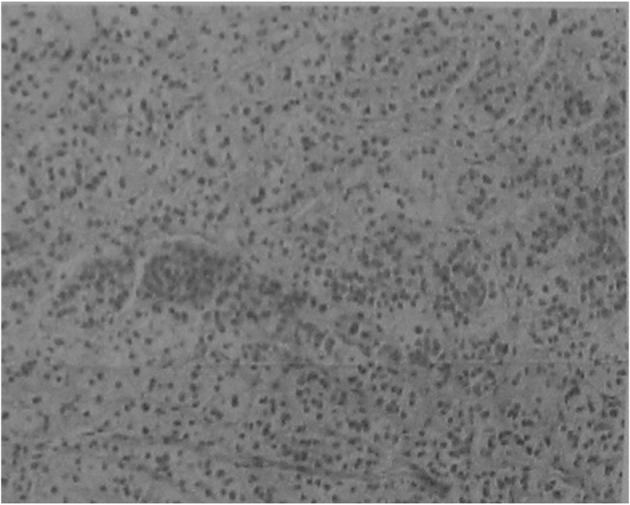
Histopathological analysis of the resected adrenal specimen.

**Figure 3:**
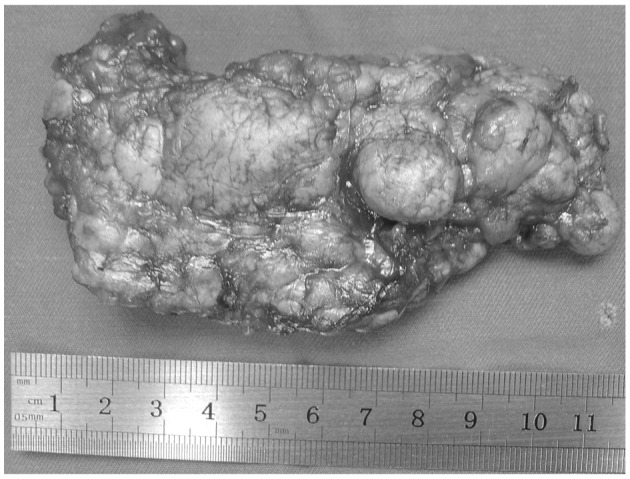
Macroscopic appearance of the adrenal glands of a patient.

Following surgery, all the resected samples were confirmed positive by histopathological analysis (Fig. 2). The nodules were observed as bright cells under the light microscope and the normal cortical structure had disappeared. The general clinical and biochemical conditions of the patients are summarized in Table II. Lumbar open surgery was performed in nine patients, retroperitoneal laparoscopic surgery was performed in seven patients, single abdominal open surgery were performed in three patients, multiple lumbar open surgery was performed in two patients and laparoscopic surgery was performed in two patients. Infection occurred in one patient following surgery, which was controlled following treatment. The resected adrenal nodular mass in all the patients was a diffused grayish yellow or golden yellow color (Fig. 3). The largest nodular was 6×4×4 cm, weighing 40 g and with no clear coat covering the resected adrenal tissue. The surface of the resected slide was golden brown and had a diameter of 0.6–1.7 cm. Patients with unilateral resection were not treated with hormone drug therapy at discharge; however, bilateral resection patients received 5 or 10 mg prednisone treatment.

### Postoperative follow-up

Following surgery, hypertension of the patients was significantly alleviated. The eight patients who underwent bilateral adrenalectomy were followed-up for 2–8 years and were treated with glucocorticoid replacement therapy. The patients had normal blood pressure, breathing rate returned to the normal level, and the obesity and sanguine appearance was alleviated.

Decreased blood pressure following surgery was observed in 15 patients. However, after three years, 12 of the 15 patients had hypertension of ~150–170/90–100 mmHg, which was maintained at 130–150/70–85 mmHg following oral administration of antihypertensive drugs. The additional three patients (cases 11, 12 and 23) had no response to oral antihypertensive drugs and their blood pressure reached 170–180/90–110 mmHg. Plasma cortisol, UFC, ACTH and CT analyses were performed, and the contralateral adrenal gland was found to have increased to 35, 32 and 38 ml, respectively. The blood pressure of these patients returned to normal following the removal of the contralateral adrenal gland and hormone replacement therapy. Nelson’s syndrome was not observed following therapy.

## Discussion

CS is caused by excessive cortisol secretion and is associated with increased mortality and severe morbidity. The condition is not fully reversible, despite biochemical control. CS is characterized by the loss of normal feedback regulation and circadian rhythm of the hypothalamic-pituitary axis due to inappropriate secretion of ACTH from a pituitary tumor (Cushing’s disease) or an ectopic source (ectopic ACTH secretion). The remaining causes (20%) are ACTH independent. Once a diagnosis is established, the therapeutic goal is the removal of the tumor. Whenever surgery is not curative, the management of patients with CS requires a major effort to control hypercortisolemia and the associated symptoms (10).

The diagnosis of CS is based on the clinical features of hypercortisolism, the absence of serum cortisol diurnal rhythm, elevated midnight sleeping cortisol levels and incomplete cortisol suppression test (11). CS due to AIMAH was first reported in an isolated case (12).

AIMAH is characterized by bilateral macronodular hyperplasia of the adrenal glands and is one of the causes of CS (13). Clinical manifestations include hypertension, weight gain, impaired glucose tolerance or diabetes mellitus, osteoporosis and an increased susceptibility to bruising. Hypogonadism and gynecomastia have been reported in males and hirsutism in females (14). The precise etiology of AIMAH is unknown; however, previous studies have demonstrated that aberrant adrenal expression and aberrant function of a number of peptide hormone receptors, including receptors for glucose-dependent insulinotropic hormone, vasopressin, luteinizing hormone/human chorionic gonadotropin, β-adrenergic agonists and serotonin, may lead to adrenal cell proliferation and abnormal regulation of steroidogenesis in AIMAH (15,16).

Cross-sectional imaging is commonly used to identify adrenal disease in patients with CS. CT and MRI scans are used to document the lesion size and shape, presence or absence of calcification, hemorrhage and necrosis. With regard to MRI, T1-weighted images are hypointense relative to the liver and isointense relative to muscle, while T2-weighted images tend to be hyperintense relative to the liver (17,18). By contrast, the nodules of patients with chronic ACTH stimulation appear isointense relative to the liver on T2-weighted MRI scans (19).

Histological analysis revealed a marked increase in the number of small clear cells, which are predominantly derived from the upper fascicular zone. The amount of cortisol produced by each cell is small; thus, significant enlargement of the adrenal gland is necessary before excessive cortisol production causes CS (20). The definitive treatment of CS consists of surgical resection of the tumor secreting ACTH. When the source of excessive cortisol secretion is the pituitary gland, the standard approach is to perform an endoscopic endonasal trans-sphenoidal exploration, with excision of the tumor if identified. This surgical procedure is demanding and should only be performed in centers with extensive experience to minimize the surgical risks, reduce the possibility of remission and maintain other pituitary gland functions.

Bilateral adrenalectomy via an overt or laparoscopic approach is the most useful treatment for patients with AIMAH and hormonal hypersecretion (21,22). However, in patients exhibiting moderately increased hormonal production, unilateral adrenalectomy is proposed as a safe and effective alternative; it is expected that as the cell mass increases in the contralateral adrenal gland, a second adrenalectomy may be required (23,24).

In conclusion, a total of 23 cases diagnosed with AIMAH were presented in the current study. High and low dose dexamethasone failed to suppress cortisone secretion in the suppression tests and ACTH levels were low in all the cases. Bilateral enlarged adrenal glands were observed on CT scans, and bilateral adrenal macronodular hyperplasia was confirmed in all the cases by pathological examination. In accordance with previous studies (25,26), the results of the present study demonstrated that AIMAH had unique endocrinological, radiological and pathological features. Diagnosis of AIMAH is predominantly derived from pathological examination and long term remission may be achieved by unilateral adrenalectomy. Contralateral adrenalectomy should be performed in cases of recurrence, when followed with periodical examination of the symptoms and serum concentration of cortisol.

## Figures and Tables

**Table I:** Levels of plasma cortisol, UFC and ACTH in 23 patients with AIMAH.

				Prior to surgery			Nodule volume^a^ (ml)		Three years following surgery		
						
Patient	Age (years)	Gender	Examination time	Cortisol (nmol/l)	UFC (nmol/24 h)	ACTH (pmol/l)	Left	Right	Cortisol (nmol/l)	UFC (nmol/24 h)	ACTH (pmol/l)
1	36	M	8 am	718	1684	0.2	14	18	-	-	-
			0 am	828	-	-	-	-	-	-	-
			8 pm	1132	-	-	-	-	-	-	-
2	42	M	8 am	883	1601	0.1	30	32	-	-	-
3	47	F	8 am	800	1341	0.6	27	18	497	400	-
4	39	M	8 am	938	1311	0.9	14	14	580	428	-
5	57	F	8 am	2070	1203	0.4	60	32	-	-	-
6	52	F	8 am	828	828	0.1	32	14	-	-	-
7	55	M	8 am	469	359	0.7	27	27	276	304	-
8	51	M	8 am	552	2622	0.7	30	14	414	386	-
			0 am	552	-	-	-	-	-	-	-
			8 pm	331	-	-	-	-	-	-	-
9	61	M	8 am	994	1573	0.6	32	50	524	320	-
10	59	F	8 am	1104	1159	0.7	38	30	-	-	-
11	47	M	8 am	1214	966	1.0	50	16	966	657	0.9
12	48	M	8 am	1711	1242	0.9	55	18	1104	980	0.9
13	53	F	8 am	1065	1423	1.2	50	26	450	380	1.8
			0 am	855	-	-	-	-	-	-	-
			8 pm	686	-	-	-	-	-	-	-
14	48	M	8 am	960	630	0.7	45	45	500	350	0.8
15	46	F	8 am	350	520	0.8	40	32	260	170	1.1
			0 am	440	-	-	-	-	-	-	-
			8 pm	460	-	-	-	-	-	-	-
16	47	M	8 am	900	1420	0.9	33	42	430	290	2.1
17	39	M	8 am	840	1125	1.2	46	28	410	260	2.0
18	48	M	8 am	880	900	1.3	40	33	-	-	-
19	59	F	8 am	1156	1236	0.5	45	48	650	780	0.9
20	42	M	8 am	710	840	1.1	38	36	430	390	1.2
21	45	F	8 am	850	960	0.8	40	38	600	620	1.0
22	57	M	8 am	910	1022	0.7	45	40	710	510	0.8
23	49	F	8 am	950	1150	0.9	50	29	580	420	1.2

Cortisol reference value, 138–690 nmol/l; UFC reference value, 27.6–276 nmol/24 h; ACTH reference value, 2.2–22 pmol/l. AIMAH, ACTH-independent macronodular adrenal hyperplasia; UFC, urinary free cortisol; ACTH, adrenocorticotropic hormone; M, male; F, female.

a Nodule volume was measured post-surgery.

**Table II:** General clinical and biochemical conditions of the patients following surgery.

Case	Resected position	Blood loss (ml)	Resected adrenal gland weight (L/R/g)	Discharge time following surgery (days)	Discharge medication	Complication	Surgical method
1	R	250	−/36	9	-	-	Lumbar open surgery
3	L	200	54/−	11	-	-	Lumbar open surgery
4	L	150	28/−	11	-	-	Lumbar open surgery
6	L	250	64/−	9	-	-	Lumbar open surgery
7	R	300	−/54	11	-	-	Lumbar open surgery
8	L	250	60/−	14	-	-	Lumbar open surgery
11	L	250	100/−	11	-	-	Lumbar open surgery
12	L	200	110/−	10	-	-	Lumbar open surgery
13	L	150	100/−	10	-	-	Lumbar open surgery
15	L	350	80/−	9	-	-	Retroperitoneal laparoscopic surgery
16	R	150	−/84	12	-	-	Retroperitoneal laparoscopic surgery
17	L	100	92/−	13	-	-	Retroperitoneal laparoscopic surgery
18	L	90	80/−	11	-	-	Retroperitoneal laparoscopic surgery
20	L	90	76/−	11	-	-	Retroperitoneal laparoscopic surgery
23	L	100	100/−	12	-	-	Retroperitoneal laparoscopic surgery
2	Bi	350	60/64	21	Prednisone (5 mg, bid)	-	Retroperitoneal laparoscopic surgery
5	Bi	400	120/64	20	Prednisone (5 mg, bid)	-	Single abdominal open surgery
10	Bi	400	76/60	19	Prednisone (10 mg, bid)	-	Single abdominal open surgery
9	Bi	450	64/100	21	Prednisone (5 mg, bid)	-	Single abdominal open surgery
14	Bi	510	90/90	19	Prednisone (10 mg, bid)	-	Multiple lumbar open surgery
19	Bi	250	90/96	20	Prednisone (10 mg, bid)	-	Multiple lumbar open surgery
21	Bi	150	80/76	21	Prednisone (5 mg, bid)	-	Laparoscopic surgery
22	Bi	200	90/80	30	Prednisone (5 mg, bid)	Infection	Laparoscopic surgery

R, right; L, left; Bi, bilateral.
